# Molecular mechanism of calcium induced trimerization of C1q-like domain of otolin-1 from human and zebrafish

**DOI:** 10.1038/s41598-021-92129-8

**Published:** 2021-06-17

**Authors:** Rafał Hołubowicz, Andrzej Ożyhar, Piotr Dobryszycki

**Affiliations:** Department of Biochemistry, Molecular Biology and Biotechnology, Faculty of Chemistry, Wrocław University of Science and Technology, Wybrzeże Wyspiańskiego 27, 50-370 Wrocław, Poland

**Keywords:** Calcium, Metalloproteins, Chromatography, Protein purification, PCR-based techniques, Circular dichroism, Fluorescence spectroscopy

## Abstract

The C1q superfamily includes proteins involved in innate immunity, insulin sensitivity, biomineralization and more. Among these proteins is otolin-1, which is a collagen-like protein that forms a scaffold for the biomineralization of inner ear stones in vertebrates. The globular C1q-like domain (gC1q), which is the most conserved part of otolin-1, binds Ca^2+^ and stabilizes its collagen-like triple helix. The molecular details of the assembly of gC1q otolin-1 trimers are not known. Here, we substituted putative Ca^2+^-binding acidic residues of gC1q otolin-1 with alanine to analyse how alanine influences the formation of gC1q trimers. We used human and zebrafish gC1q otolin-1 to assess how evolutionary changes affected the function of the protein. Surprisingly, the mutated forms of gC1q otolin-1 trimerized even in the absence of Ca^2+^, although they were less stable than native proteins saturated with Ca^2+^. We also found that the zebrafish gC1q domain was less stable than the human homologue under all tested conditions and became stabilized at higher concentrations of Ca^2+^, which showed that specific interactions leading to the neutralization of the negative charge at the axis of a gC1q trimer by Ca^2+^ are required for the trimers to form. Moreover, human gC1q otolin-1 seems to be optimized to function at lower concentrations of Ca^2+^, which is consistent with reported Ca^2+^ concentrations in the endolymphs of fish and mammals. Our results allow us to explain the molecular mechanism of assembly of proteins from the C1q superfamily, the modulating role of Ca^2+^ and expand the knowledge of biomineralization of vertebrate inner ear stones: otoliths and otoconia.

## Introduction

Balance sensing in vertebrates depends on signals received in the inner ear, specifically in the semicircular canals and in the vestibule. The semicircular canals allow the detection of angular acceleration thanks to the inertial flow of the endolymph, a fluid that fills the inner ear, which deflects the cupula and thus stimulates mechanoreceptors in the sensory epithelium. The vestibule consists of an utricle, a saccule, and in all vertebrates besides mammals, of a lagena, which are collectively called vestibular organs. The vestibular organs contain biominerals called “ear stones” anchored to the sensory epithelium through a gelatinous membrane^1,2^. They contain calcium carbonate (90–97% by mass), biological macromolecules including glycoproteins and proteoglycans and trace mineral additives, especially Sr^2+^ and Ba^2+^^3–6^. The ear stones have a higher density than the endolymph, and during movement of the head, they move relative to the sensory epithelium, which allows to sense linear acceleration. They also contribute to spatial orientation due to the sensing of the gravitational field^2^. The ear stones differ among vertebrates. In general, in fish, they form large continuous crystals called otoliths; in terrestrial vertebrates, they appear as tiny multiple otoliths, specifically named otoconia, which are embedded in a gelatinous membrane^3,7^. These biominerals contain calcium carbonate of different polymorphs. Most often, teleost fish otoliths contain aragonite or vaterite^8^. Otoconia can contain aragonite (amphibians), calcite (birds and mammals), or a mixture of both polymorphs (reptiles)^7^. More detailed reviews of the evolution of otoliths and otoconia can be found elsewhere^3,9^. The mechanism of biomineralization of otoliths and otoconia, especially the mechanism of polymorph selection, is poorly understood^10,11^.

Numerous studies have shown that biomineralization of otoliths and otoconia is controlled by proteins, many of which are acidic and bind calcium ions (Ca^2+^)^11^. For example, intrinsically disordered Starmaker protein (Stm) is necessary to form properly shaped aragonite otoconia in zebrafish *Danio rerio*^12^. The biomineralization activity of Stm and its analogues from other fish was thoroughly analysed in vitro^13–16^, but although these proteins strongly influenced the size and shape of calcium carbonate crystals grown in vitro, they were not sufficient to induce the formation of aragonite instead of calcite, the most stable polymorph of calcium carbonate. In the case of otolith matrix macromolecule-64 (OMM-64) from rainbow trout *Oncorhynchus mykiss*, an analogue of Stm, aggregated otolith matrix proteins were indeed shown to be required together with OMM-64 to form aragonite in vitro^17^. Recently, transcriptomic and proteomic studies have allowed to identify proteins that constitute complex protein mixtures of the endolymph and otolith matrix^16,18^. Remarkably, a major constituent of the aggregate is otolin-1—a short chain collagen-like protein, which is strongly conserved in evolution^16,18,19^. Proteolytic cleavage of otolin-1 has been suggested to be a key step in temporally regulated deposition of aragonite in otoliths^18^.

The term “otolin” was first used in the 1960s for a component of the otolith matrix, which maintained similar amino acid compositions among different species of fish^20^. In the 2000s, genes encoding otolin-1 from various organisms were cloned^21–24^, and many more otolin-1 genes since then have been identified on the basis of homology. Otolin-1 belongs to the C1q superfamily of proteins, which includes short chain collagenous proteins involved in a wide array of biological processes such as innate immune response (C1q)^25^, insulin sensitivity (adiponectin)^26^, cell adhesion (collagen VIII, CTRP5)^27,28^ and biomineralization (collagen X, otolin-1)^29,30^. In addition to a collagen-like domain, otolin-1 contains a short N-terminal domain rich in cysteine residues and a globular C1q-like (gC1q) domain at the C-terminus. Function of the N-terminal domain is not known. In adiponectin, a serum protein hormone similar to otolin-1, the N-terminal fragment is responsible for the aggregation of adiponectin trimers to biologically active high molecular weight aggregates^26,31,32^. The gC1q domain is inherently able to form trimers that, together with the ability of the collagen-like domain to form a triple helix, enable it to form fibrils, which could be interconnected by N-terminal domains and gC1q domains. Such a fibrillary network could provide a scaffold for biomineralization of otoliths and otoconia^11,30,33^. Moreover, murine otolin-1 has been shown to interact with otoconin-90 (Oc90), a major otoconial matrix protein, through the collagen-like and gC1q domains^24,34^. This interaction enhances the biomineralization activity of otolin-1 and Oc90^30^, indicating that otolin-1 has two critical functions in the biomineralization of otoliths and otoconia, namely, structural function by providing a scaffold for biomineralization and regulatory function by interacting with other matrix proteins, which allows biomineralization to be controlled. The affinity for Ca^2+^ also suggests the direct involvement of otolin-1 in the localized precipitation of calcium carbonate through interactions with the mineral phase^33^.

The molecular mechanism of otolin-1 trimerization is not known. Similar proteins from the C1q superfamily such as collagen X, adiponectin or C1q contain Ca^2+^ in their gC1q domains near the axes of the trimers^29,35,36^. In this work, we substituted two conserved acidic residues of the gC1q domain of human and zebrafish otolin-1 (abbreviated as hOtolC1q and dOtolC1q, respectively), which may bind Ca^2+^, with neutral alanine, and we analysed the influence of mutations on the effect of Ca^2+^ on the secondary structure, oligomerization propensity and stability of gC1q otolin-1. We also checked whether an abundant natural missense polymorphism in the human otolin-1 gene impacts the structure of the protein. Single mutations of the putative Ca^2+^-binding residues caused mixed effects. Surprisingly, when both residues were mutated, trimers of gC1q domains were highly stable even in the absence of Ca^2+^. Nevertheless, from all studied variants and conditions, native forms of the gC1q domain of otolin-1 saturated with Ca^2+^ were the most stable. We observed significant differences between hOtolC1q and dOtolC1q. A single mutation in the calcium binding site was enough to diminish the ability of dOtolC1q to bind Ca^2+^, which was not the case for hOtolC1q. Moreover, dOtolC1q and its mutants had lower thermal stability than all variants of hOtolC1q in all tested conditions. These observations allow us to propose a mechanism of oligomerization of otolin-1, in which the neutralization of the negative charge by Ca^2+^ is required at the axis of the gC1q trimer.

## Results

### Predictions indicate detrimental effects of mutations in the gC1q domain of otolin-1

**Figure 1:**
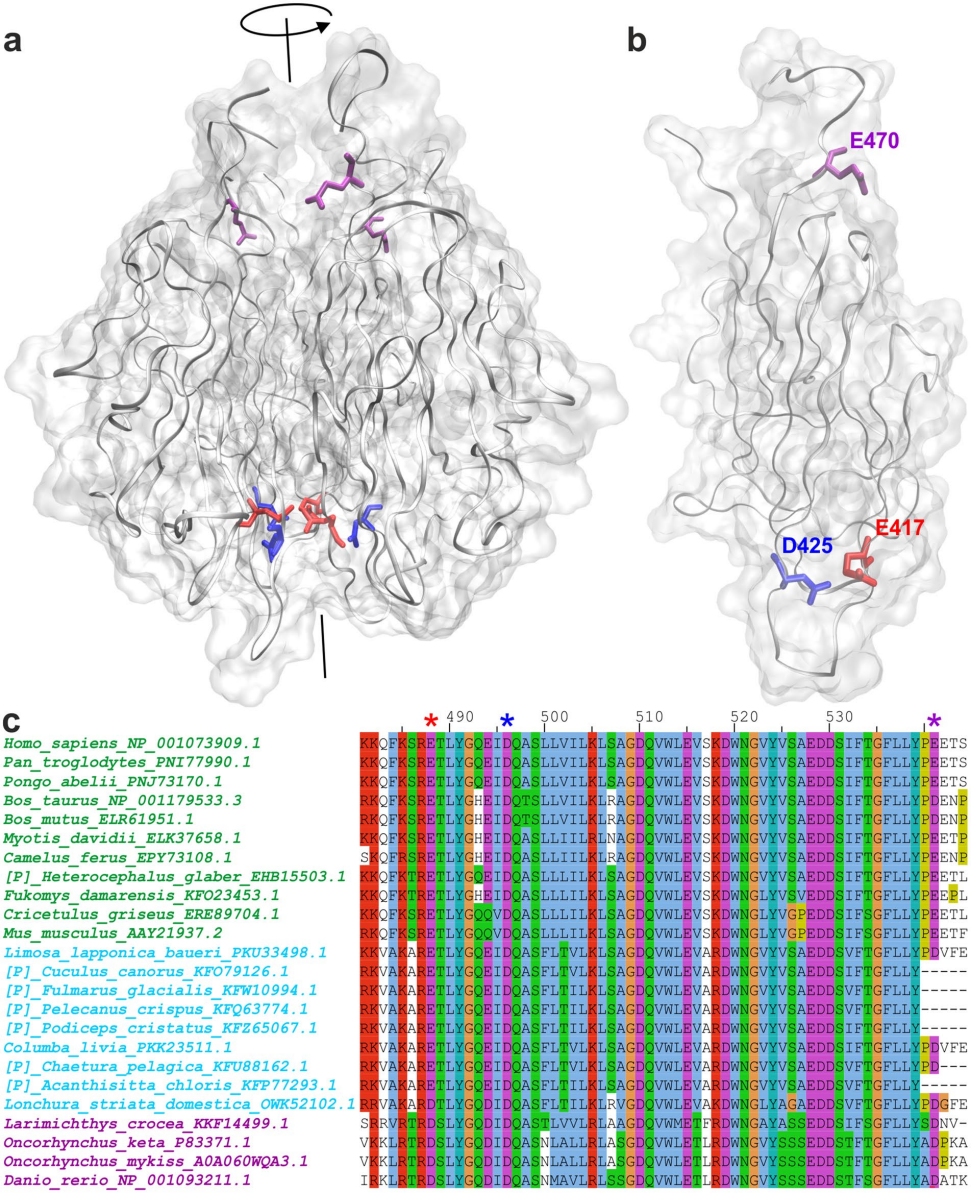
Molecular model of the gC1q domain of otolin-1 from human (hOtolC1q) and zebrafish (dOtolC1q). (**a**, **b**) Model of hOtolC1q (**a**) trimer and (**b**) monomer obtained through EOM analysis based on small angle X-ray scattering data^33^. The predicted shape of the molecule is shown in light grey, the protein backbone is shown as grey ribbons, E417 is shown in red, D425 in dark blue, and E470 in purple. Axis of a trimer is shown in black. The images were prepared using VMD software (University of Illinois, https://www.ks.uiuc.edu/Research/vmd/)^65^. (**c**) Part of a multiple sequence alignment of otolin-1 sequences from mammals (green), birds (blue) and fish (purple), including E417 (red asterisk), D425 (dark blue asterisk) and E470 (purple asterisk) residues of hOtolC1q. Annotations on the left include organism names and NCBI gene identifiers. [P] indicates partial protein sequences. The alignment is coloured using Clustal colour scheme. Briefly, the colours mark at least partially conserved residues. Blue indicates hydrophobic residues, red—basic, magenta—acidic, green—polar, cyan—aromatic, orange—glycines, yellow—prolines, pink—cysteines (absent in the analysed fragment). A more detailed description is available at https://www.jalview.org/help/html/colourSchemes/clustal.html. The alignment was performed using Clustal X^66^ and visualized using Jalview^67^.

We used data acquired from the Ensembl database and predictions in silico to screen currently known missense single nucleotide polymorphisms (SNPs) of hOtolC1q and dOtolC1q for their abundance and possible effect on protein function and stability. To be qualified as an SNP, a variant must be found in at least 1% of the population^37^. Following this definition, we found a single SNP in a region coding hOtolC1q, in which glutamate-470 is replaced by alanine (E470A) due to a substitution of adenine with cytosine (variant ID: rs3921595). The prevalence of this variant is close to 50%. Such a widespread genetic variant should not have any adverse effect on human health. However, in the model of hOtolC1q obtained through ensemble optimization method (EOM) analysis of small angle X-ray scattering (SAXS) data^33^, E470 is predicted to be near the trimerization interface (Fig. 1a,b). Therefore, E470 could be engaged in the stabilization of a gC1q trimer through ionic interactions. The substitution of glutamate with alanine at this position might impair the folding of otolin-1 into a trimer or decrease its stability by inducing aggregation. The importance of E470 of otolin-1 is highlighted by a strong conservation of this residue among different organisms (Fig. 1c, purple asterisk). The damage potential of the E470A substitution is indicated by SIFT and PolyPhen2; however, the model-based SNP MuSiC tool predicts this variant to be neutral (Table 1). We decided to analyse how E470A impacts the solution structure of hOtolC1q and its sensitivity towards Ca^2+^.

**Table 1:** Predicted effects of mutations of hOtolC1q and dOtolC1q.

Mutant	SIFT score	SIFT prediction	PolyPhen2 score	PolyPhen2 prediction	SNP MuSiC score	SNP MuSiC prediction
hOtolC1q E417A	0.03	Deleterious	0.98	Probably damaging	0.60	Deleterious
hOtolC1q D425A	0.01	Deleterious	1.00	Probably damaging	0.79	Deleterious
hOtolC1q E470A	0.04	Deleterious	0.63	Possibly damaging	− 0.15	Neutral
dOtolC1q D432A	0.02	Deleterious	0.99	Probably damaging	0.71	Deleterious
dOtolC1q D440A	0.00	Deleterious	0.99	Probably damaging	0.81	Deleterious

The predictions were made using SIFT (https://sift.bii.a-star.edu.sg/)^62^, PolyPhen2 (http://genetics.bwh.harvard.edu/pph2/)^63^ and SNPMuSiC (https://soft.dezyme.com/)^64^.

SIFT score has a scale from 0 to 1. Variants with scores below 0.05 are predicted to be deleterious.

PolyPhen2 score has a scale from 0 to 1. Variants with scores up to 0.446 are predicted to be benign, from 0.447 to 0.908 to be possibly damaging, and with scores higher than 0.908 to be probably damaging.

SNPMuSiC: positive score predicts the variant to be deleterious, negative to be neutral.

Acidic residues that contribute to the binding of Ca^2+^ by the gC1q domains of collagen X and adiponectin are also strongly conserved in otolin-1 (Fig. 1c, red and blue asterisks). Therefore, in addition to testing the effect of the E470A substitution in hOtolC1q, we decided to analyse how substitution of putative Ca^2+^-binding aspartate (hOtolC1q D425, dOtolC1q D432 and D440) and glutamate (hOtolC1q E417) residues with alanine would affect hOtolC1q and dOtolC1q. Mutations of the putative Ca^2+^ binding sites of hOtolC1q and dOtolC1q are clearly predicted to be damaging to the protein by all tools used (Table 1).

### Tb^3+^ as a LRET probe for binding of Ca^2+^

**Figure 2:**
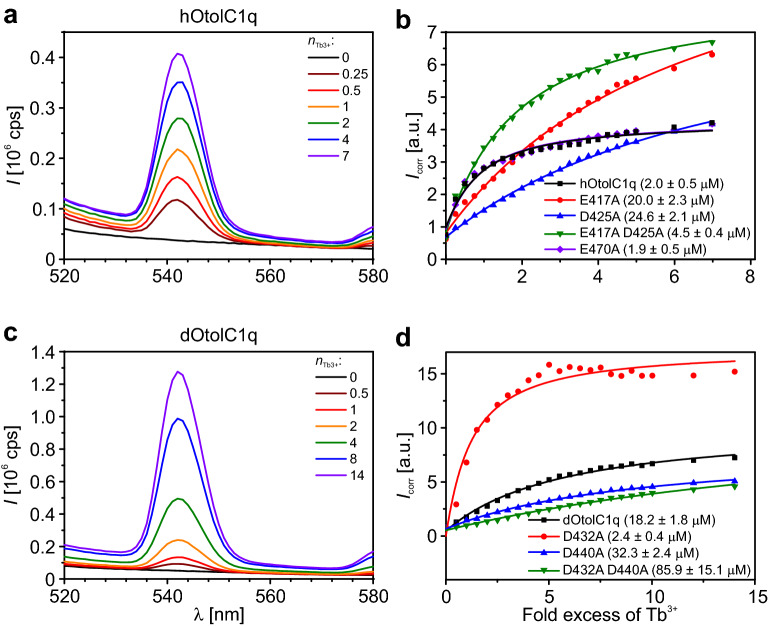
Binding of Tb^3+^ by hOtolC1q, dOtolC1q and their mutants. (**a**) Effect of Tb^3+^ (molar fold excess indicated as n_Tb3+_) on the emission spectrum of hOtolC1q excited at 280 nm. (**b**) Binding curves obtained for hOtolC1q and its mutants. (**c**) Effect of Tb^3+^ on the emission spectrum of dOtolC1q excited at 280 nm. (**d**) Binding curves obtained for dOtolC1q and its mutants. Apparent dissociation constants are given in parentheses. Protein concentration was 3.7 µM in all experiments. After adding aliquots of TbCl_3_ stock, the samples were incubated for 15 min before measuring the fluorescence emission intensity (*I*) from 520 to 580 nm at 1 nm/s. For calculations, the fluorescence intensity was standardized (*I*_corr_) by dividing *I*_545_ by *I*_530_^70^. Apparent dissociation constants were calculated using a model of a single Tb^3+^ binding site per gC1q otolin-1 monomer with Origin Pro software.

We attempted to measure Ca^2+^ binding properties of native and mutated hOtolC1q and dOtolC1q. However, upon the removal of Ca^2+^ by overnight incubation with buffered Chelex 100 resin (Bio-Rad, Hercules, CA, USA)^38^, native hOtolC1q and dOtolC1q precipitated completely. Dialysis against Ca^2+^-depleted buffer did not result in satisfactory removal of bound Ca^2+^. Upon concluding that the proteins were not obtainable in a Ca^2+^-free form without the addition of EDTA or EGTA, we decided to indirectly estimate the affinity of the proteins to Ca^2+^ by measuring the binding of terbium(III) ions (Tb^3+^), as Ca^2+^-binding proteins can also bind lanthanide ions due to similar effective ionic radii and positive charges of Ca^2+^ and lanthanide ions^39^. The binding of lanthanide ions by Ca^2+^-binding proteins is usually stronger than the binding of Ca^2+^, due mainly to a higher positive charge^40–42^. Therefore, protein-bound Ca^2+^ should be substituted by Tb^3+^. Specifically, the use of Tb^3+^ in such studies gives a significant advantage, as when bound to proteins, Tb^3+^ can be detected thanks to luminescence resonance energy transfer (LRET) from aromatic side chains (phenylalanine, tryptophan and tyrosine) to the bound Tb^3+^. Upon excitation of the proteins at the aromatic absorption band (280 nm), sensitized emission from the bound Tb^3+^ can be observed with a maximum at 545 nm^39,43^. Both hOtolC1q and dOtolC1q contain numerous aromatic residues, which are a prerequisite for LRET to occur. The results of the titration experiments are shown in Fig. 2.

When the samples of hOtolC1q and dOtolC1q contained Tb^3+^, fluorescence emission in the visible part of the spectrum with a maximum at 545 nm was observed upon excitation at 280 nm. The intensity of fluorescence emission increased when subsequent portions of TbCl_3_ were added (Fig. 2a,c). Apparent dissociation constants (*K*_d_) calculated using a model of a single binding site per protein monomer were 2.0 µM for hOtolC1q and 18.2 µM for dOtolC1q (Fig. 2b,d). Mutants of hOtolC1q with at least one putative Ca^2+^ binding residue mutated bound Tb^3+^ weaker than the native protein, whereas the double mutant hOtolC1q E417A D425A (*K*_d_ = 4.5 µM) seemed to bind Tb^3+^ stronger than E417A (*K*_d_ = 20.0 µM) or D425A (*K*_d_ = 24.6 µM) mutants. E470A substitution is in this case indistinguishable from native hOtolC1q. In the case of dOtolC1q, the D440A mutant bound Tb^3+^ weaker than dOtolC1q (*K*_d_ = 32.3 µM). Surprisingly, the D432A mutant (*K*_d_ = 2.4 µM) seemed to have a higher affinity for Tb^3+^ than native dOtolC1q (*K*_d_ = 18.2 µM). A double mutant dOtolC1q D432A D440A had the lowest affinity to Tb^3+^ of all tested forms of dOtolC1q (*K*_d_ = 85.9 µM).

Mutations of the putative Ca^2+^-binding residues did not abolish the ability of OtolC1q to bind Tb^3+^. For all mutants except dOtolC1q D432A and hOtolC1q E470A, the affinity was decreased compared to the affinity of the native proteins, as indicated by an increase in *K*_d_. The E470 residue of hOtolC1q did not seem to bind Tb^3+^, as substitution with alanine did not affect the affinity of hOtolC1q towards Tb^3+^. In the case of dOtolC1q D432A, we noted an increase in affinity (decrease in *K*_d_). Notably, the emission intensity of dOtolC1q D432A was the highest of all tested forms of OtolC1q (Fig. 2d), which indicated that the mutation caused a structural change, which displaced the aromatic residues closer to the Ca^2+^ binding site, as the efficiency of LRET increased. The structural change also seems to facilitate the binding of Tb^3+^. In further experiments, we analysed how the mutations affected the secondary structure, oligomerization propensity and thermal stability of the gC1q domain of otolin-1. The experiments were conducted in the presence of EDTA, Ca^2+^ and Tb^3+^ to expand the conclusions drawn from the Tb^3+^ binding experiment.

### Mutations affected the secondary structure of hOtolC1q and dOtolC1q

**Figure 3:**
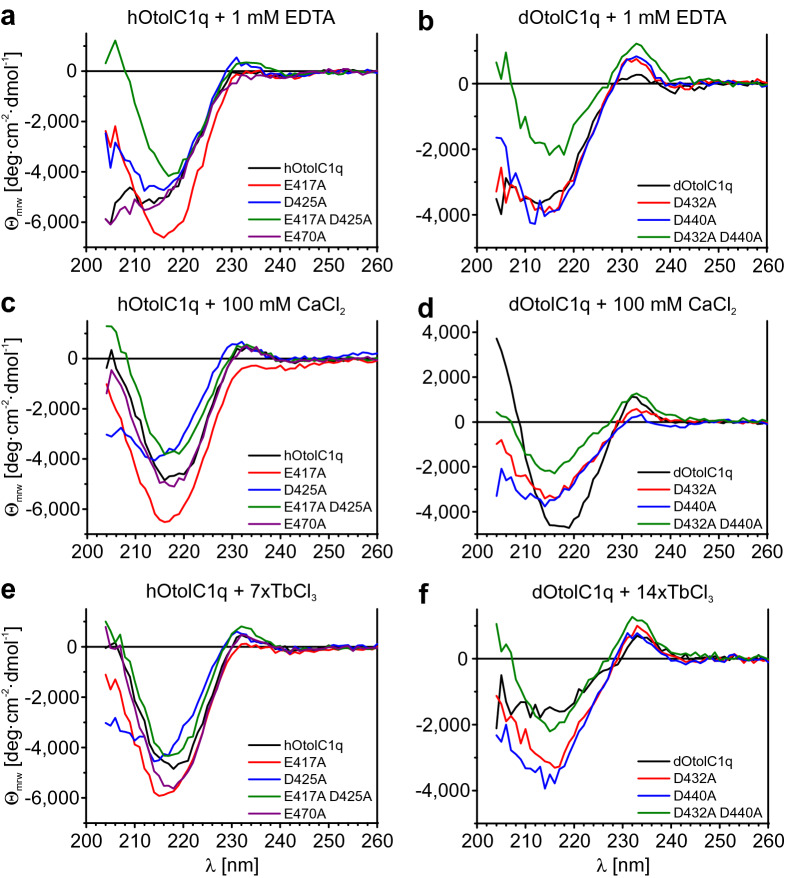
Changes in the secondary structure of hOtolC1q, dOtolC1q and their mutants caused by Ca^2+^ and Tb^3+^. Circular dichroism (CD) spectra were obtained for (**a**, **c**, **e**) hOtolC1q, (**b**, **d**, **f**) dOtolC1q and their mutants in the presence of (**a**, **b**) 1 mM EDTA, (**c**, **d**) 10 mM CaCl_2_, (**e**) sevenfold molar excess of TbCl_3_ or (**f**) 14-fold molar excess of TbCl_3_. The protein concentration was 0.2 mg/ml. The spectra are an average of 5 subsequent measurements and were corrected for the signal from a solvent. *Θ*_mrw_—mean residual ellipticity. See also Suppl. Fig. 1.

Circular dichroism spectroscopy (CD) is a useful, sensitive technique that allows us to estimate the secondary structure of proteins and to detect changes in the structure induced by denaturation, stabilization or ligand binding^44^. We have previously used it to analyse Ca^2+^-induced structural changes in hOtolC1q and dOtolC1q^33^. Here, we used CD to identify structural changes induced by the mutations. CD also allowed us to assess secondary structure changes accompanied by binding of Ca^2+^ and Tb^3+^ to mutated hOtolC1q and dOtolC1q (Fig. 3, Suppl. Fig. 1). Concentrations of TbCl_3_ chosen for the experiments were saturating for native hOtolC1q and dOtolC1q. Higher concentrations, but still as low as 1 mM TbCl_3_ (85-fold molar excess), caused visible precipitation of both hOtolC1q and dOtolC1q (not shown).

In the case of hOtolC1q, spectra collected for proteins depleted from Ca^2+^ by 1 mM EDTA indicate that mutations involving the Ca^2+^ binding site led to changes in β-structure content (Fig. 3a). Native hOtolC1q has a negative ellipticity band at approximately 215 nm, which is attributed to β-sheets, and a decreasing ellipticity below 215 nm. The 215-nm band became deeper for hOtolC1q E417A, shallower for D425A and shallowest for E417A D425A. The spectra also changed at 233 nm in the positive aromatic side chain band^45,46^. E470A substitution did not affect the CD spectrum of hOtolC1q. Spectra collected for proteins in the presence of 100 mM CaCl_2_ indicate that only the native hOtolC1q and the E470A variant significantly changed their structures upon binding of Ca^2+^ (Fig. 3c, Suppl. Fig. 1a,b,d–f). For these proteins, in the presence of 100 mM CaCl_2_, the ellipticity below 215 nm increased instead of slightly decreasing, as was observed for 1 mM EDTA. This effect can be attributed to an increase in β-structure content with a subsequent decrease in protein disorder^33^. The addition of 100 mM CaCl_2_ to hOtolC1q D425A led to the shallowing of the 215 nm negative ellipticity band, indicating a minor structural change (Suppl. Fig. 1e). The spectra collected in the presence of a sevenfold excess of Tb^3+^ are similar to the spectra collected in the presence of Ca^2+^ (Fig. 3e, Suppl. Fig. 1a,b,d–f). Slight differences in ellipticity at 215 nm were observed for the E417A, D425A and E470A variants (Suppl. Fig. 1b,d,e). It seems that the E470A variant, despite binding Tb^3+^ with similar affinity to native hOtolC1q, has a slightly different secondary structure than native hOtolC1q when Tb^3+^ ions are bound. We conclude that in terms of the secondary structure of hOtolC1q, the binding of Tb^3+^ has a similar effect as the binding of Ca^2+^.

In the case of dOtolC1q, all spectra collected in the presence of 1 mM EDTA, despite that for the D432A D440A mutant, are similar (Fig. 3b). For dOtolC1q, the negative band at approximately 215 nm indicates the presence of β-sheets. The D432A and D440A mutants can be distinguished from the native form by an increased ellipticity at the 233-nm band, which is attributed to aromatic residues. This difference supports the conclusion that aromatic residues were displaced due to the mutations, and thus, the LRET signal was strongly increased for D432A and slightly decreased for D440A. Weakening of the CD signal of dOtolC1q D432A D440A when compared to other forms suggests that the double mutation of the Ca^2+^ binding site interrupted folding of dOtolC1q. When the spectra collected in the presence of 1 mM EDTA were compared with those obtained for samples with 100 mM CaCl_2_, it became apparent that the D440A and D432A D440A mutants were not sensitive to the presence of Ca^2+^ (Suppl. Fig. 1 h,i). As observed previously^33^, dOtolC1q underwent a major structural change when Ca^2+^ was added (Fig. 3, Suppl. Fig. 1c). The ellipticity minimum at 215 nm becomes deeper, and the signal strongly increases below 215 nm. As for hOtolC1q, this is interpreted as induction of β-sheets from the disordered portions of the protein. The structural change is more pronounced for dOtolC1q than for hOtolC1q. A weaker but significant change in the spectrum was also observed for dOtolC1q D432A (Suppl. Fig. 1g). This indicates that the D432A mutant retained some affinity towards Ca^2+^. The spectrum of dOtolC1q collected in the presence of a 14-fold molar excess of Tb^3+^ is much shallower below 230 nm than in the presence of 1 mM EDTA or 100 mM CaCl_2_. We interpret such a major change in the spectrum as a manifestation of destabilization of the protein, as at this concentration of protein (0.2 mg/ml, 11.7 µM) and Tb^3+^ (163.8 µM), precipitation became visible after prolonged incubation. The protein precipitated immediately at higher concentrations of dOtolC1q and Tb^3+^. For the mutants, the spectra collected in the presence of Tb^3+^ were similar to those collected in the presence of Ca^2+^. We conclude that, contrary to hOtolC1q, the binding of Ca^2+^ to dOtolC1q cannot be reliably simulated using Tb^3+^. Nevertheless, we can conclude that the double mutants (hOtolC1q E417A D425A and dOtolC1q D432A D440A) are completely unable to bind Ca^2+^, as their secondary structure is the same in the presence of 1 mM EDTA, 100 mM CaCl_2_ and an appropriate excess of TbCl_3_.

### Mutations affected Ca^2+^-dependent trimerization of hOtolC1q and dOtolC1q

**Figure 4:**
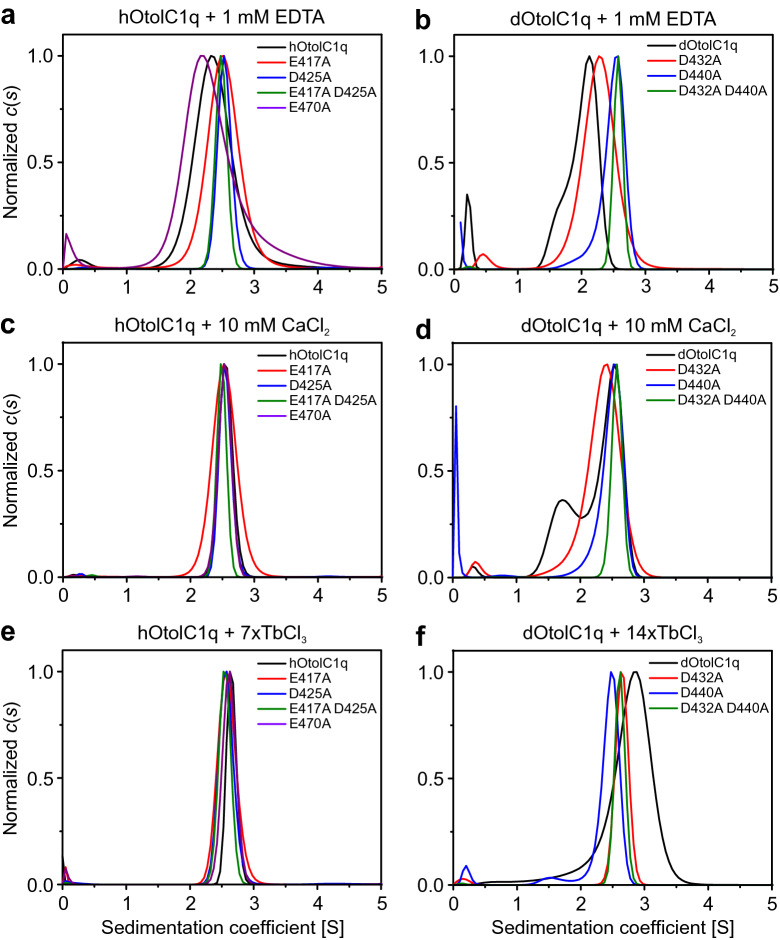
Normalized sedimentation coefficient distributions (*c*(*s*)) of hOtolC1q, dOtolC1q and their mutants in the presence of Ca^2+^ and Tb^3+^. The results of sedimentation velocity analytical ultracentrifugation (SV AUC) experiments for (**a**, **c**, **e**) hOtolC1q, (**b**, **d**, **f**) dOtolC1q and their mutants in the presence of (**a**, **b**) 1 mM EDTA, (**c**, **d**) 10 mM CaCl_2_, (**e**) sevenfold molar excess of TbCl_3_ or (**f**) 14-fold molar excess of TbCl_3_. The protein concentration was in the range of 0.20–0.25 mg/mL. SV AUC data were fitted to a continuous sedimentation coefficient distribution (*c*(*s*)) model using SEDFIT software. The plots were standardized for a maximal *c*(*s*) peak in the range 0.5–4.5 S. See also Suppl. Fig. 2, Suppl. Fig. 3 and Suppl. Table 1.

The CD measurements showed significant secondary structure changes induced by mutations in the Ca^2+^ binding sites of hOtolC1q and dOtolC1q. However, analysis of the influence of Tb^3+^ on the secondary structure of hOtolC1q and dOtolC1q and comparison with the effect of Ca^2+^ gave inconclusive results. It seems that Tb^3+^ can be used to simulate the binding of Ca^2+^ to hOtolC1q but not to native dOtolC1q, which aggregates in the presence of Tb^3+^. We analysed the tertiary structures of hOtolC1q, dOtolC1q and their mutants using sedimentation velocity analytical ultracentrifugation (SV AUC) to see how mutations of Ca^2+^ binding sites affected the oligomerization propensity of gC1q otolin-1. We hypothesized that as Ca^2+^ stabilizes trimers of hOtolC1q and dOtolC1q^33^, alanine mutations in the Ca^2+^ binding site could affect the formation of gC1q trimers. We also checked the influence of Ca^2+^ and Tb^3+^ on the structure of the mutants to see how the eventual changes correspond to the results of LRET and CD measurements. The results of SV AUC are summarized in Fig. 4, Suppl. Fig. 2, Suppl. Fig. 3 and Suppl. Table 1.

Introduced mutations had a significant impact on the oligomerization propensity of hOtolC1q and dOtolC1q. (Fig. 4a,b). In the presence of 1 mM EDTA, hOtolC1q and dOtolC1q formed unstable oligomeric forms, which sedimented slower than expected trimers due to the fast association and dissociation events occurring during the overnight ultracentrifugation experiment^33^. Alanine mutations in the Ca^2+^ binding site induced stable oligomerization even in the absence of Ca^2+^, as evidenced by the apparent molecular weight values (*MW*_app_) derived from SEDFIT analysis (Suppl. Table 1), which indicated that hOtolC1q D425A, hOtolC1q E417A D425A and dOtolC1q D432A D440A form stable trimers even in the presence of 1 mM EDTA (monomer *MW* is 17.0 kDa for hOtolC1q and 17.1 kDa for dOtolC1q). This observation was unexpected, as a decrease in trimerization propensity was anticipated to occur due to the introduced mutations. hOtolC1q E417A also seems to form trimers in the absence of Ca^2+^, but a wider *c*(*s*) distribution than for D425A and E417A D425A mutants (Fig. 4a) suggests the lower stability of the oligomer. dOtolC1q D440A seems to form trimers, but a trace of lower molecular weight forms is present (Fig. 4b). dOtolC1q and dOtolC1q D432A did not form stable oligomers, but the *c*(*s*) distribution of dOtolC1q D432A compared to native dOtolC1q seems to be skewed towards heavier forms. Analysis of the concentration dependence of *c*(*s*) distributions (Suppl. Fig. 3, Suppl. Table 1) shows that the trimers formed by hOtolC1q with any of the Ca^2+^-binding residues mutated are stable, which is also true for dOtolC1q D432A D440A, with both Ca^2+^-binding residues being mutated. Both the D432A and D440A mutants of dOtolC1q show concentration dependence of *c*(*s*) distributions, but it is not as strong as in the case of dOtolC1q. When the proteins were analysed in the presence of 10 mM CaCl_2_, it became apparent that the oligomers formed by the dOtolC1q D432A mutant were not as stable as the oligomers formed by native dOtolC1q (Fig. 4c,d). Under these conditions, dOtolC1q formed dimers and trimers. The proportion of trimers relative to dimers increased with increasing concentrations of dOtolC1q (Suppl. Fig. 3c, Suppl. Table 1). In contrast, the D432A mutant still did not form stable trimers. The *c*(*s*) distributions reveal a small concentration dependence (Suppl. Fig. 3g). Mutating the D432 residue of dOtolC1q seemed to disturb the formation of stable trimers. Neither dOtolC1q D440A nor the double mutant seemed to be affected by Ca^2+^ (Suppl. Fig. 2h,i; Suppl. Fig. 3h,i). Sedimentation profiles of hOtolC1q E417A, D425A and a double mutant also did not change upon the addition of Ca^2+^.

When Tb^3+^ ions were added to the proteins instead of Ca^2+^, hOtolC1q and dOtolC1q did not respond in the same way (Fig. 4e,f). hOtolC1q and all its mutants formed stable trimers in the presence of Tb^3+^ (Fig. 4e). This is different for dOtolC1q because its *c*(*s*) distribution shows signs of aggregation at 0.25 mg/ml protein and 14-fold molar excess of TbCl_3_ (Fig. 4f), amplified by the observation from preliminary experiments, in which 0.5 mg/ml dOtolC1q mixed with 1 mM TbCl_3_ precipitated completely. (i.e., the absorbance signal collected during SV AUC was zero, data not shown). In the presence of Tb^3+^, dOtolC1q D432A finally formed stable trimers. *c*(*s*) distributions of dOtolC1q D440A and dOtolC1q D432A D440A did not change compared to the *c(s)* distributions calculated for proteins with EDTA or Ca^2+^ (Suppl. Fig. 2h,i). The mutated forms of dOtolC1q seem to be stable in the presence of Tb^3+^ in a trimeric form, even though the native protein was not.

Compared to native hOtolC1q, the *c*(*s*) distribution of the natural variant hOtolC1q E470A in the absence of Ca^2+^ has a maximum at a lower sedimentation coefficient and a tail at a higher sedimentation coefficient (Fig. 4 a, Suppl. Fig. 3a,b), which may suggest decreased trimerization propensity and increased tendency to form aberrant aggregates, but similar sedimentation coefficients for both forms indicate that the effect is weak (Suppl. Table 1). In the presence of Ca^2+^ and Tb^3+^, both forms have highly similar *c*(*s*) distributions. Under these conditions, both the hOtolC1q and E470A variants formed stable trimers without any trace of unbound subunits or heavier aggregates (Fig. 4c,e).

SV AUC provided interesting information about the effects of mutations of the Ca^2+^ binding site of gC1q otolin-1. The substitution of conserved Ca^2+^ binding aspartate and glutamate residues with alanine seems to diminish the ability to bind Ca^2+^, which is normally found at the axis of the gC1q trimer^29,47,48^. However, instead of inhibiting Ca^2+^-dependent formation of the trimers, this substitution led to the formation of stable trimers even in the absence of Ca^2+^. This effect may be explained by the neutralization of the potentially repulsive electrostatic charge at the axis of the gC1q trimer by substitution of negatively charged carboxyl groups of aspartate and glutamate with uncharged methyl groups of alanine. Only in the case of dOtolC1q, mutation of D432 with D440 left intact clearly disturbed the Ca^2+^-dependent trimerization of the protein. Interestingly, Tb^3+^ allowed this mutant to form trimers. The modified Ca^2+^ binding site of dOtolC1q D432A seems to be accidentally optimized for the binding of Tb^3+^.

### Mutations decreased the stabilizing effect of Ca^2+^ on hOtolC1q and dOtolC1q

**Figure 5:**
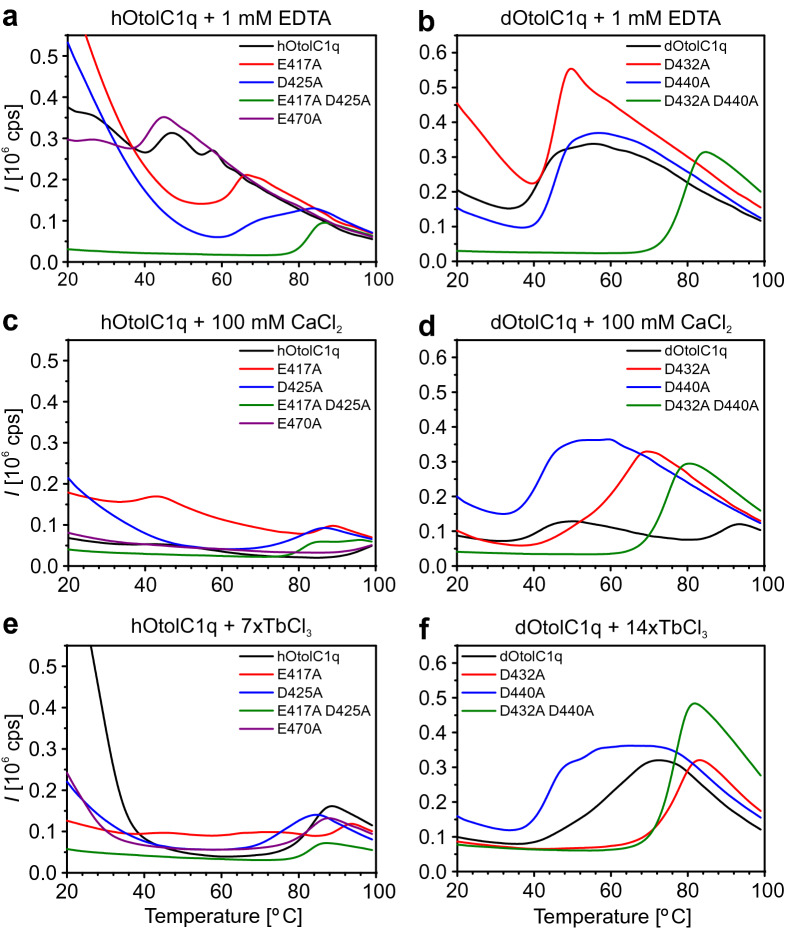
Thermal stability of hOtolC1q, dOtolC1q and their mutants in the presence of Ca^2+^ and Tb^3+^. The results of differential scanning fluorimetry (DSF) experiments for (**a**, **c**, **e**) 5 µM hOtolC1q, (**b**, **d**, **f**) 5 µM dOtolC1q and their mutants in the presence of (**a**, **b**) 1 mM EDTA, (**c**, **d**) 100 mM CaCl_2_, (**e**) sevenfold molar excess of TbCl_3_ and (**f**) 14-fold molar excess of TbCl_3_. The experiment was conducted in the presence of a 10X concentration (500-fold dilution of a 5000 × stock solution) of SYPRO Orange in an ImageQuant Studio 5 qPCR thermal cycler with heating at 0.033 °C/s. All samples were measured in triplicate, and representative denaturation profiles are shown.

hOtolC1q and dOtolC1q were stabilized by Ca^2+^, which was shown using CD and differential scanning fluorimetry (DSF) with SYPRO Orange as a probe^33^. In this work, we used DSF to analyse how the mutations affected the thermal stability of hOtolC1q and dOtolC1q. We also analysed the effect of Ca^2+^ and Tb^3+^ on the stability of the mutants. Both hOtolC1q and dOtolC1q were strongly stabilized by Ca^2+^ (Fig. 5, Table 2), and their transition temperature (*T*_m_) increased from 41 to 44 °C in the absence of Ca^2+^ to 90 °C or more at 100 mM CaCl_2_ (Fig. 5, Table 2). Interestingly, mutants of hOtolC1q with substituted Ca^2+^-binding residues were more stable than native hOtolC1q in the absence of Ca^2+^; their *T*_m_ values were 62–65 °C for E417A and D425A and as high as 83 °C for the double mutant. hOtolC1q E470A had a denaturation profile very similar to hOtolC1q under all tested conditions. Ca^2+^ stabilized the E417A and D425A mutants, as their *T*_m_ was above 80 °C in 100 mM CaCl_2_. The double mutant, E417A D425A, did not seem to be affected by Ca^2+^ up to 10 mM CaCl_2_. In 100 mM CaCl_2_, the double mutant was slightly destabilized. Although hOtolC1q with any of the Ca^2+^ binding residues mutated was more stable than the native form in the absence of Ca^2+^, native hOtolC1q supplemented with 100 mM CaCl_2_ was the most stable of all samples measured, also true for the E470A variant, in which the Ca^2+^ binding site was unaffected. Although this concentration of Ca^2+^ seems to be very high compared to the physiological Ca^2+^ concentration in the mammalian endolymph (approximately 0.1 mM)^49^, it simulates intracrystalline conditions of otoconia. Tb^3+^ ions stabilized hOtolC1q and all its mutants, but the effect was not the same as the effect for Ca^2+^.

**Table 2:** Thermal stability of hOtolC1q, dOtolC1q and their mutants in the presence of Ca^2+^ and Tb^3+^.

Ligand	*T*_m_ [°C]								
	hOtolC1q	E417A	D425A	E417A D425A	E470A	dOtolC1q	D432A	D440A	D432A D440A
1 mM EDTA	43.7	62.5	65.6	82.7	41.2	41.6	45.5	45.4	79.5
0.1 mM CaCl_2_	44.5; 57.1	69.8	78.5	83.0	41.9	41.8	46.4	45.9	80.1
1 mM CaCl_2_	66.2	76.4	77.0	83.0	66.7	41.4	46.8	46.2	79.8
10 mM CaCl_2_	85.9	83.6	78.3	82.7	86.8	41.0	50.8	45.6	79.2
100 mM CaCl_2_	97.3	39.0; 85.7	80.7	80.9	97.2	41.1; 89.3	64.9	41.8	74.9
7/14 × TbCl_3_	83.8	90.5	74.8	86.9	83.1	58.2	77.9	44.5	76.7

The transition temperatures (*T*_m_) of hOtolC1q, dOtolC1q and their mutants in the presence of 1 mM EDTA, 0.1–100 mM CaCl_2_ and an appropriate molar excess of TbCl_3_ (7 × for hOtolC1q, 14 × for dOtolC1q) were measured using differential scanning fluorimetry (DSF). *T*_m_ was determined from the derivative of fluorescence with increasing temperature (dF/dT).

In the case of dOtolC1q, the D432A and D440A mutants were slightly more stable than dOtolC1q, as their *T*_m_ was approximately 45 °C in the absence of Ca^2+^. The double mutant D432A D440A was very stable under these conditions, as its *T*_m_ reached 80 °C. The addition of Ca^2+^ stabilized dOtolC1q and, to a lesser extent, the D432A mutant. Native dOtolC1q and the D432A mutant were also stabilized by Tb^3+^, but the effect was different than for 100 mM CaCl_2_: weaker for dOtolC1q and stronger for D432A. Stabilization of dOtolC1q D432A by Tb^3+^ relative to Ca^2+^ is another indication that the mutation changed the structure of the protein, resulting in preferential binding of Tb^3+^. Strikingly, D440A and the double mutant D432A D440A were not stabilized by Ca^2+^. Similar to hOtolC1q E417A D425A, they were destabilized by 100 mM CaCl_2_. These mutants were also not stabilized by Tb^3+^. We can therefore conclude that hOtolC1q E417A D425A, dOtolC1q D440A and dOtolC1q D432A D440A are completely unable to bind Ca^2+^ or Tb^3+^ at the mutated binding site. The fluorescence signal observed in Tb^3+^ binding experiments for these mutants may come from nonspecific binding of Tb^3+^ at adventitious sites^40^, seemingly without an effect on their thermal stability. Mutation of a single residue, D440, seems to be sufficient to diminish the ability of dOtolC1q to bind Ca^2+^. An analogous mutant of hOtolC1q, D425A, seems to retain minor affinity to Ca^2+^.

## Discussion

In this work, a set of biophysical techniques was used to assess the mechanism of trimerization and the influence of mutations of chosen acidic residues on the structure and Ca^2+^ binding properties of hOtolC1q and dOtolC1q. The results are summarized in Table 3. We observed interesting differences between human and zebrafish forms of the gC1q domain of otolin-1, as a single mutation (D440A) was enough to completely diminish the ability of dOtolC1q to bind calcium ions. Although the single mutations at the Ca^2+^ binding site of hOtolC1q (E417A and D425A) strongly decreased its affinity to Ca^2+^, the mutants were still stabilized by Ca^2+^ and Tb^3+^, as evidenced by DSF. The double mutant (hOtolC1q E417A D425A) was not stabilized by these ions. All tested forms of hOtolC1q had notably higher thermal stability than the respective forms of dOtolC1q under all tested conditions. Moreover, hOtolC1q was stabilized at a lower concentration of Ca^2+^ than dOtolC1q, clearly showing that during evolution, the gC1q domain of otolin-1 became optimized to function at lower concentrations of Ca^2+^. Endolymph Ca^2+^ concentration in teleosts varies, as it was reported to be 1.0 mM in turbot^50^, 1.0–1.6 mM in trout^50–52^ and as high as 2.9 mM in oyster toadfish^53^. In mammals, endolymph Ca^2+^ concentration was determined to be much lower, between 92 and 133 µM, depending on the compartment (guinea pig)^49^.

**Table 3:** Summary of the effects of the mutations on the structure of hOtolC1q and dOtolC1q and their interactions with Ca^2+^.

	Secondary structure changed by the mutation?	Secondary structure changed by the addition of Ca^2+^?	Stable trimers in the absence of Ca^2+^?	Stable trimers in the presence of Ca^2+^?	Δ*T*_m_ by 100 mM Ca^2+^ versus 1 mM EDTA [°C]
hOtolC1q	–	Yes	No	Yes	53.6
E417A	Yes	No	Yes	Yes	23.2
D425A	Yes	No	Yes	Yes	15.1
E417A D425A	Yes	No	Yes	Yes	− 1.8
E470A	No	Yes	No	Yes	56
dOtolC1q	–	Yes	No	Yes	47.7
D432A	Yes	Yes	No	No	19.6
D440A	Yes	No	Yes	Yes	− 3.6
D432A D440A	Yes	No	Yes	Yes	− 4.6
Data in	Fig. 2	Fig. 3, Suppl. Fig. 1	Fig. 4, Suppl. Fig. 2, Suppl. Table 1,1		Fig. 5, Table 2

In silico predictions for the prevalent natural polymorphism E470A, involving a residue that was modelled to be away from the Ca^2+^ binding site of hOtolC1q, were ambiguous in terms of the effect on protein stability. Thermal stability in the presence of Ca^2+^, binding of Ca^2+^ or Tb^3+^ and solution structure of hOtolC1q were not significantly affected by this substitution. The only notable effects were decreased thermal stability of the E470A mutant below 1 mM Ca^2+^ and increased tendency to aggregate in the absence of Ca^2+^. Normally, this effect would have been negligible in vivo. Otolin-1, as a secreted protein, is after translation exposed to the ER lumen, which contains 100–800 µM Ca^2+^^54^. This concentration should be sufficient to stabilize the gC1q domain of the E470A variant. In the utricle and saccule, otolin-1 could be protected from low concentrations of Ca^2+^ by being bound within otoconia and cross-linked with other proteins in the otoconial membrane. However, a decrease in local Ca^2+^ caused by dysregulated calcium homeostasis or degeneration of otoconia might destabilize the protein, possibly accelerating the degradation of otoconial matrix and/or otoconial membrane. Therefore, it would be interesting to examine whether the presence of the E470A allele in the genome is associated with the prevalence of benign paroxysmal positional vertigo (BPPV). BPPV is a balance disorder caused by displacement of degraded otoconial membrane fragments to the semicircular canals^55^, where they disrupt the flow of endolymph or movement of cupula, which occur normally due to the movement of the head. This effect is felt by the affected person as sudden attacks of vertigo, which may lead to falls and bone fractures and, consequently, to disability, especially later in life^56,57^. The hypothesized lability at low Ca^2+^ is consistent with our observation that hOtolC1q E470A is particularly difficult to produce in *Escherichia coli*. The E470A variant is the sole form of hOtolC1q studied here that required the addition of a detergent to remain soluble after cell lysis, and still the purification yield was much lower than the purification yield for native hOtolC1q. The concentration of Ca^2+^ in the cytoplasm of *E. coli*, which was shown to be in the range of 0.3–1.6 µM^58^, is too low to stabilize hOtolC1q E470A. In the context of recombinant protein production, an increased tendency to form aggregates at low Ca^2+^ would be especially detrimental, as it would facilitate the precipitation of the protein in the form of inclusion bodies^59^. E470 of human otolin-1 is conserved in fish. If such a mutation would have a similar effect on piscine otolin-1, the continuous growth of the otoliths could be disrupted in the event of catastrophic loss of endolymph Ca^2+^. However, as native dOtolC1q was stabilized at higher Ca^2+^ than hOtolC1q, such an event would likely also affect the fish not having this mutation.

Mutations of the conserved Ca^2+^-binding residues of hOtolC1q and dOtolC1q were, however, consistently predicted to be deleterious and functionally damaging for the protein. Our results show that the predictions were not fully correct. All forms of hOtolC1q and dOtolC1q with any residue from the Ca^2+^ binding site mutated formed more stable oligomers in the absence of Ca^2+^ than the native proteins. Significant deleterious effects of the mutations became apparent when the proteins were studied with 10–100 mM CaCl_2_. Under these conditions, the D432A mutant of dOtolC1q was unable to form stable trimers, contrary to native dOtolC1q. Interestingly, this mutant seemed to be accidentally optimized for the binding of Tb^3+^ relative to Ca^2+^, as the mutation increased the apparent affinity of the protein towards Tb^3+^. This mutant also formed stable trimers only in the presence of Tb^3+^ and not in the presence of Ca^2+^. The most important observation, however, is that although the mutation of the Ca^2+^ binding sites of hOtolC1q or dOtolC1q seemed to have a stabilizing effect, native hOtolC1q and dOtolC1q were the most thermally stable forms in the conditions simulating the interiors of otoliths and otoconia. Additionally, hOtolC1q E417A, hOtolC1q E470A and all mutants of dOtolC1q were difficult to handle in the laboratory: the expression yields were smaller than for the native forms, and protein loss during purification was significantly higher. The mutants were also difficult to concentrate. The highest achievable concentration of hOtolC1q was 4 mg/mL, and of dOtolC1q was 1.5 mg/mL. In contrast, hOtolC1q E417A and all mutants of dOtolC1q began to precipitate at approximately 0.5 mg/mL.

Crystallographic data show that Ca^2+^ is bound at the axis of a gC1q domain trimer^29,48^. Mutation of the Ca^2+^-binding residues of the gC1q domain of C1q-like protein 3 (C1QL3) significantly decreased its thermal stability^48^. It was plausible to conclude that mutation of a Ca^2+^ binding site in the gC1q domain of otolin-1 could disrupt the formation of trimers due to the lack of a stabilizing effect of Ca^2+^. However, mutations of the acidic residues forming the Ca^2+^ binding site to alanine actually facilitated the trimerization of hOtolC1q and dOtolC1q, explained by the neutralization of the repulsive negative charge at the trimerization interface by replacing acidic residues with uncharged residues. Interestingly, a gC1q domain of collagen VIII also forms trimers in the absence of Ca^2+^^27^. In this case, the Ca^2+^ binding sites are occupied by side chains of lysine residues from the same subunits, also leading to neutralization of the negative charge. We conclude that Ca^2+^-dependent neutralization of a negative charge at the Ca^2+^-binding site is crucial for the formation of the gC1q domain trimer.

Although our data do not explain why an increased preference of dOtolC1q D432A for Tb^3+^ occurs, the binding of Tb^3+^ is known not to be fully equivalent to the binding of Ca^2+^, although these ions have similar ionic radii due to the higher charge and higher observed coordination number of Tb^3+^ complexes compared to Ca^2+^ complexes with proteins^40,60,61^. Moreover, changes in hydrogen bonding around the metal binding site were observed when lanthanide ions were bound instead of Ca^2+^ to compensate for the charge difference^40^. In our case, this inequivalence apparently leads to the formation of stable dOtolC1q D432A trimers in the presence of Tb^3+^ but not in the presence of Ca^2+^, possibly also the reason for severe destabilization of dOtolC1q in the presence of Tb^3+^.

In light of the results of Tb^3+^-binding experiments, which seem to bind strongly to and thermally stabilize hOtolC1q, dOtolC1q and their mutants, it is surprising that the proteins were actually destabilized if we consider their solubility. For dOtolC1q, strong immediate precipitation in the presence of Tb^3+^ was observed at 0.5 mg/mL protein. During prolonged incubation, some precipitation was also observed at 0.2 mg/mL. Human isoforms seemed to better tolerate the presence of Tb^3+^. In the LRET experiment, Tb^3+^ seemed to bind to mutants that were not responsive to Ca^2+^. In the case of dOtolC1q, this did not lead to thermal stabilization of its nonbinding mutants. Tb^3+^ ions may have been bound away from the Ca^2+^ binding sites. Such “adventitious” binding of lanthanide ions at sites that do not bind Ca^2+^ was observed in the crystal structures of proteins and was characterized by a lower coordination number. The remaining coordination sites of lanthanide ions were speculated to be occupied by water^40^. In the case of dOtolC1q, the available coordination sites of Tb^3+^ could possibly become occupied by functional groups from other trimers and lead to the formation of heavy aggregates of dOtolC1q. Weak binding of Tb^3+^ at the adventitious sites could result in aggregation occurring at a relatively high protein concentration, above 0.2 mg/mL.

In summary, we discovered that the basis of the engagement of Ca^2+^ in the formation of gC1q domain trimers is the neutralization of the negative charge at the Ca^2+^ binding site. Despite the apparently stabilizing effect of substitutions of acidic Ca^2+^-binding residues with neutral alanine residues, hOtolC1q and dOtolC1q were ultimately the most stable in their native forms saturated with Ca^2+^. We also determined that hOtolC1q and its mutants were more stable than dOtolC1q and its mutants under all tested conditions, including a Ca^2+^-free environment. Apparently, the human protein became optimized to function at lower concentrations of Ca^2+^. We conclude that this predisposes otolin-1 to years-long function in biomineral organic matrices.

## Methods

### Accession numbers

Human *OTOL1* gene Ensembl accession ID: ENSG00000182447.

Zebrafish *OTOL1A* gene Ensembl accession ID: ENSDARG00000001771.

Human otolin-1 UniProt accession ID: A6NHN0.

Zebrafish otolin-1 UniProt accession ID: A5PN28.

Human otolin-1 E470A SNP variant ID: rs3921595.

### Key resources

Synthetic cDNAs encoding full-length otolin-1 from human and zebrafish were codon-optimized for *E. coli* and provided by GeneArt (currently Thermo Fisher Scientific, Warsaw, Poland). Nucleotide primers were provided by Genomed (Warsaw, Poland). The pQE-80 L plasmid expression vector was purchased from Qiagen (Hilden, Germany). *E. coli* Top10 cells, Phusion DNA polymerase, restriction enzymes, T4 DNA ligase and LB broth were obtained from Thermo Fisher Scientific. One-fusion DNA polymerase was obtained from GeneOn (Ludwigshafen am Rhein, Germany; distributed by ABO, Gdańsk, Poland). Agar, agarose, tris(hydroxymethyl)aminomethane (Tris), ethylenediaminetetraacetic acid (EDTA), carbenicillin, isopropyl β-d-1-thiogalactopyranoside (IPTG), NaCl, glycerol, 2-mercaptoethanol, imidazole, glycine, sodium dodecyl sulfate (SDS) and CaCl_2_ were obtained from Carl Roth (Karlsruhe, Germany; distributed by Linegal Chemicals, Warsaw, Poland). *E. coli* BL21(DE3) cells, TB broth, 4-(2-hydroxyethyl)-1-piperazineethanesulfonic acid (HEPES), lysozyme, phenylmethylsulfonyl fluoride (PMSF), DNase I, RNase A, terbium(III) chloride hexahydrate, xylenol orange disodium salt and SYPRO Orange were from Sigma (currently Merck, Warsaw, Poland). The NP40 substitute was from Amresco (currently VWR, Gliwice, Poland). Empty Tricorn, Superdex 200 Increase 10/300 GL and Superdex 75 16/60 Prep Grade columns were obtained from GE Healthcare Life Sciences (currently Cytiva, Warsaw, Poland). TALON® Metal Affinity resin and 6xHis monoclonal antibody (albumin-free) were purchased from Takara Bio (Mountain View, CA, USA; distributed by Biokom, Janki, Poland). HRP horse anti-mouse IgG antibody was purchased from Vector Laboratories (Burlingame, CA, USA; distributed by Biokom, Janki, Poland).

### Identification of single nucleotide polymorphisms

The Ensembl genome browser (www.ensembl.org/) was queried for known single nucleotide polymorphisms (SNPs) in otolin-1 genes. The gene identifiers were ENSG00000182447 (*OTOL1*) for human otolin-1 and ENSDARG00000001771 (*OTOL1A*) for zebrafish otolin-1. Boundaries of gC1q domains were retrieved from the UniProt database (A6NHN0 for human otolin-1 and A5PN28 for zebrafish otolin-1) as 338–477 for human and 351–489 for zebrafish otolin-1.

### Prediction of mutation effects

Natural and designed mutants were assessed using online tools to predict their effects on protein structure and function. Data for the natural E470A SNP accompanied a respective entry in the Ensembl database, from which the results for SIFT (https://sift.bii.a-star.edu.sg/)^62^ and PolyPhen2 (http://genetics.bwh.harvard.edu/pph2/)^63^ tools were chosen for comparisons, as these tools were also useful to predict the effects of artificial mutations for both hOtolC1q and dOtolC1q. Additionally, for all investigated mutations, the SNP MuSiC (https://soft.dezyme.com/) tool was used to predict the effects on protein stability^64^. Models of gC1q trimers, which were used as a template, were based on already published EOM analysis conducted on the basis of SAXS data^33^. Default parameters were used in all predictions. The structure model was visualized using VMD software (University of Illinois, https://www.ks.uiuc.edu/Research/vmd/)^65^. Sequences of otolin-1 from selected fish, birds and mammals were aligned using Clustal X^66^ and visualized using Jalview^67^.

### Preparation of mutated gC1q genes

Genes coding the mutants of hOtolC1q: E417A, D425A and E417A D425A and of dOtolC1q: D432A, D440A and D432A D440A were prepared by amplifying the synthetic cDNAs of hOtolC1q and dOtolC1q in two parts using Phusion DNA polymerase. PCR products were digested using the *Esp*3I (*Bsm*BI) restriction enzyme, which cleaves DNA 5 bp away from the recognition site. Cohesive ends of the cleavage products contained codons, which introduced the mutations. The gene fragments were subcloned into the pQE-80 L vector using *Bam*HI and *Hind*III restriction sites. In the case of hOtolC1q E470A, synthetic cDNA of hOtolC1q subcloned into the pQE-80 L plasmid expression vector^33^ was used as a template in modified QuickChange®, which was conducted as described^68^. One-fusion DNA polymerase was used in the mutagenic PCR. For the calculation of the annealing temperatures of the primers, the concentration of KCl in the reaction mixture was assumed to be 0.1 M, as in the assay buffer of the polymerase. Plasmids were propagated in *E. coli* TOP10 cells. The progress of the cloning was followed by agarose gel electrophoresis. All mutated genes were analysed by DNA sequencing (Genomed).

### Protein expression and purification

*Escherichia coli *BL21(DE3) cells were chemically transformed by heat shock and grown overnight on plates containing LB broth with 1.5% agar and 100 µg/mL carbenicillin at 37 °C. Single colonies were picked and used to inoculate starter cultures containing 100 ml of TB broth with carbenicillin, which were incubated overnight at 37 °C and 200 rpm. Portions of 500 ml TB with carbenicillin were inoculated with a 2% volume of starter culture and incubated at 29 °C and 200 rpm. After reaching the optical density at 600 nm of 0.5, cultures were cooled to 15 °C, and the expression of the protein of interest was induced by 0.5 mM IPTG. The culture was continued overnight (16–18 h) at 15 °C and 200 rpm. Cells were collected by centrifugation at 5000×*g* at 4 °C for 15 min and resuspended in H10Na500G5 buffer [HEPES 10 mM, pH 7.0 (20 °C), NaCl 500 mM, glycerol 5% (v/v)] with 1 mM 2-mercaptoethanol and 1 mg/ml lysozyme. Suspensions of cells expressing hOtolC1q E470A were additionally supplemented with 0.1% NP40 substitute (Amresco, currently VWR, Gliwice, Poland). The cells were kept frozen at − 80 °C.

Cell lysis was initiated by thawing in a room-temperature water bath. After thawing, 0.2 mg/mL PMSF, 20 µg/mL DNase I and 20 µg/mL RNase A were added. Lysis was facilitated by 10 cycles of ultrasonic treatment for 30 s with 1-min breaks in a Cole-Parmer CPX 500 ultrasonic processor with a microtip and amplitude set at 35% (Cole-Parmer, Vernon Hills, IL, USA). The cell suspension was kept on ice to maintain the temperature below 10 °C. Lysates were clarified by centrifugation at 18,500×*g* for 30 min at 4 °C and incubated with 1 mL bed volume of TALON® Metal Affinity resin for 1 h in a cold room (4–6 °C) in an orbital mixer set at 5 rpm. The resin was separated by centrifugation at 700×*g* for 5 min at 4 °C, washed with 20 bed volumes of H10Na500G5, centrifuged again and packed in a glass Tricorn column. The column was connected to ÄKTA Avant chromatography system (GE Healthcare Life Sciences) with a flow rate of 1 mL/min. Contaminants were washed away with 20 bed volumes of H10Na500G5 and subsequently with 20 bed volumes of the buffer with 30 mM imidazole. The protein of interest was eluted with buffer containing 200 mM imidazole. The eluate was concentrated in Amicon Ultra centrifuge filters with a 10 kDa cut-off (Merck) and subjected to gel filtration using a Superdex 200 Increase 10/300 GL column operated at 0.75 mL/min (hOtolC1q and its mutants) or Superdex 75 16/60 Prep Grade operated at 1.5 mL/min (dOtolC1q and its mutants) with H10Na500G5 as a mobile phase. Eluate before void volume of the column was collected for use as a reference in further studies. Fractions containing proteins were collected and analysed using SDS-PAGE with an acrylamide percentage of 4% in a stacking gel and 12% in a resolving gel in a Laemmli buffer system (Tris–glycine-SDS)^69^. The identity of the protein was confirmed by Western blotting with murine 6xHis monoclonal IgG as the primary antibody and HRP horse anti-mouse IgG antibody as the secondary antibody. Pure protein samples were stored at − 80 °C.

### Tb^3+^ binding fluorescence

The binding of Tb^3+^ ions to hOtolC1q, dOtolC1q and their mutants was assessed using steady-state fluorescence. Terbium(III) chloride was dissolved in Milli-Q water to a final concentration of approximately 0.5 M. The exact concentration of TbCl_3_ was determined by titration of diluted stock solution with EDTA in the presence of xylenol orange. Aliquots of diluted TbCl_3_ were added to 2 ml 3.7 µM protein solution in a 10 × 10 mm quartz cuvette and incubated for 15 min at room temperature. Subsequently, fluorescence emission at 520–580 nm was recorded using an excitation wavelength of 280 nm using a Fluorolog-SPEX fluorimeter (HORIBA Scientific, Jobin–Yvon, Kyoto, Japan) equipped with a Peltier temperature control accessory set at 20 °C. The bandwidth was set at 5 nm for both excitation and emission monochromators. The fluorescence intensities obtained were processed and fitted to a model based on work by Gonzalez et al.^70,71^. Fluorescence intensity was corrected for sample dilution and background fluorescence using the following equation:

1 $${I}_{corr}=\frac{{I}_{max}}{{I}_{max-15}} \cdot \frac{{v}_{0}+{v}_{i}}{{v}_{0}}$$

where, *I*_corr_—corrected fluorescence, *I*_max_—fluorescence at emission maximum (here: 545 nm), *I*_max–15_—fluorescence at local emission minimum (here: 530 nm), *v*_0_—initial sample volume, *v*_i_—added volume of working solution of TbCl_3_.

Corrected fluorescence was fitted to a single binding site model to determine the apparent dissociation constant *K*_d_:

2 $${I}_{corr}={I}_{0}+\frac{{K}_{d}+{c}_{p}+{c}_{t}-\sqrt{{\left({K}_{d}+{c}_{p}+{c}_{t}\right)}^{2}-4 \cdot {c}_{p} \cdot {c}_{t}}}{a}$$

where, *I*_0_—corrected fluorescence intensity at 0 µM Tb^3+^ (fitted), *c*_p_—concentration of the protein, *c*_t_—concentration of Tb^3+^, *a*—proportionality constant (fitted).

Fitting of the data to the model above was conducted using Origin Pro 9.0 software (OriginLab, Northampton, MA, USA).

### Circular dichroism

Circular dichroism of 0.2 mg/mL proteins in H10Na500G5 with 1 mM EDTA, 0.1 mM CaCl_2_, 1 mM CaCl_2_, 10 mM CaCl_2_ or 100 mM CaCl_2_ was measured in 1 mm quartz SUPRASIL® cuvettes (Hellma Analytics, Müllheim, Germany) using a Jasco J-815 spectropolarimeter (Jasco, Easton, MD, USA) with a Peltier temperature control accessory set at 20 °C. The proteins were incubated with EDTA/CaCl_2_ at room temperature for at least 1 h before the measurements. The spectra were collected between 200 and 260 nm every 1 nm at a scanning speed of 50 nm/min with five accumulations. Data for which the photomultiplier voltage was below 600 V were analysed. CD spectra of the proteins were corrected for buffer background signal and normalized for protein composition and concentration using the equation below^72^:

3 $${\theta }_{mrw}=\frac{\theta \cdot MRW}{10 \cdot c \cdot l} \left[\frac{deg \cdot {{cm}^{2}}}{dmol}\right]$$

where ${\theta }_{mrw}$ is the mean residue ellipticity, $\theta$—ellipticity [degrees], *MRW*—mean residual weight of a protein [g/mol], *c*—protein concentration [g/L] and *l—*optical pathlength of a cuvette [cm].

### Analytical ultracentrifugation

Sedimentation velocity analytical ultracentrifugation (SV AUC) was conducted in a Beckman Coulter ProteomeLab XLI analytical ultracentrifuge (Beckman Coulter, Brea, CA, USA) with an An60Ti rotor and assembled cells with two-channel 12 mm charcoal filled Epon® centerpieces and quartz windows. The proteins were analysed at approximate concentrations of 0.1, 0.25 and 0.5 mg/ml in H10Na500G5 cells with 1 mM EDTA or 10 mM CaCl_2_. Assembled cells with the samples were preincubated in an ultracentrifuge for 3 h at 20 °C and then centrifuged at 50,000 rpm (approximately 200,000×*g* at the bottom of the cell) overnight. Scans of absorbance at 280 nm were collected continuously with 0.003 cm resolution. The scans representing the whole sedimentation process were time-corrected^73^ and analysed in SEDFIT (available at https://sedfitsedphat.nibib.nih.gov/) using a continuous *c*(*s*) distribution model^74^ with at least 20 points per 1 S. Partial specific volumes of the proteins, densities and dynamic viscosities of the solvents were calculated using SEDNTERP (available at http://www.jphilo.mailway.com/download.htm). Maximum entropy regularization with *p* = 0.95 was used. Simplex and Marquardt–Levenberg algorithms were used until the RMSD value converged. Among the results of the calculations were sedimentation coefficients (*s*), sedimentation coefficients corrected for water at 20 °C (s_20,w_), weight-averaged sedimentation coefficients ($\overline{{s_{{20,w}} }}$), apparent molecular weights (*MW*_app_) and frictional ratios (*f*/*f*_0_). *c*(*s*) distributions were visualized using GUSSI (available at https://www.utsouthwestern.edu/labs/mbr/software/)^75^ and Origin Pro 9.0 software.

### Differential scanning fluorimetry

Differential scanning fluorimetry assays were conducted as described^76^. Five micromolar solutions of the proteins in H10Na500G5 supplemented with 10 × SYPRO Orange (500-fold dilution of delivered stock solution) were measured in the presence of 1 mM EDTA, 0.1 mM CaCl_2_, 1 mM CaCl_2_, 10 mM CaCl_2_ or 100 mM CaCl_2_. Additionally, hOtolC1q and its mutants were measured in the presence of a sevenfold molar excess of TbCl_3_, dOtolC1q and its mutants in the presence of a 14-fold excess of TbCl_3_. Final sample volume was 20 µl. The samples were aliquoted into a 96-well plate in triplicate, covered with optically clear foil and incubated at room temperature for at least 1 h before the measurements. Fluorescence of SYPRO Orange was measured using an Applied Biosystems ImageQuant5 qPCR thermal cycler (Thermo Fisher Scientific) with optical filters set as x1 - m3 (excitation at 470 ± 15 nm, emission at 587 ± 10 nm) between 20 and 99 °C during heating at 0.033 °C/s. The data were analysed using Protein Thermal Shift software (Thermo Fisher Scientific). *T*_m_ were determined from the derivative of fluorescence with increasing temperature (d*F*/d*T*).

## Supplementary Information

Supplementary Information.

## Data Availability

The data and materials underlying this article will be shared on request to any of the corresponding authors.

## Abbreviations

BPPV: Benign paroxysmal positional vertigo
*c*(*s*): Continuous sedimentation coefficient distribution
CD: Circular dichroism spectroscopy
*f/f*_0_: Frictional ratio
DSF: Differential scanning fluorimetry
dOtolC1q: C1q-like domain of otolin-1 from zebrafish
EOM: Ensemble optimization method
EDTA: Ethylenediaminetetraacetic acid
gC1q: C1q-like domain; HEPES: 4-(2-hydroxyethyl)-1-piperazineethanesulfonic acid
hOtolC1q: C1q-like domain of otolin-1 from human
IPTG: Isopropyl β-d-1-thiogalactopyranoside
*K*_d_: Apparent dissociation constant
LRET: Luminescence resonance energy transfer
*MW*_app_: Apparent molecular weight
Oc90: Otoconin-90
OMM-64: Otolith matrix macromolecule-64
PMSF: Phenylmethylsulfonyl fluoride
*Θ*_mrw_: Mean residue ellipticity
$\overline{{s_{{20,w}} }}$: Weight-averaged sedimentation coefficient
SAXS: Small angle X-ray scattering
SDS: Sodium dodecyl sulphate
SNP: Single nucleotide polymorphism
Stm: Starmaker
SV AUC: Sedimentation velocity analytical ultracentrifugation
*T*_m_: Transition temperature
