# Supplementary material for: Molecular mechanism of calcium induced trimerization of C1q-like domain of otolin-1 from human and zebrafish
Source: Sci Rep. 2021 Jun 17;11:12778. doi: 10.1038/s41598-021-92129-8 (PMC8211825; doi:10.1038/s41598-021-92129-8)
Supplement: Supplementary file 1 — Supplementary Information. [file 41598_2021_92129_MOESM1_ESM.pdf]

Supplementary material

Molecular mechanism of calcium induced trimerization of C1q-like domain of otolin-1 from human and zebrafish

Rafał Hołubowicz*, Andrzej Ożyhar, Piotr Dobryszycki*

Department of Biochemistry, Molecular Biology and Biotechnology, Faculty of Chemistry, Wrocław University of Science and Technology, Wybrzeże Wyspiańskiego 27, 50-370 Wrocław, Poland

*- Corresponding Authors

R.H.: phone +48 71 320 63 34, e-mail rafal.holubowicz@pwr.edu.pl

P.D.: phone +48 71 320 63 32, e-mail piotr.dobryszycki@pwr.edu.pl

ORCID IDs: R.H.: 0000-0002-2555-2069; A.O.: 0000-0002-5874-784X; P.D.: 0000-0001-5522-2557

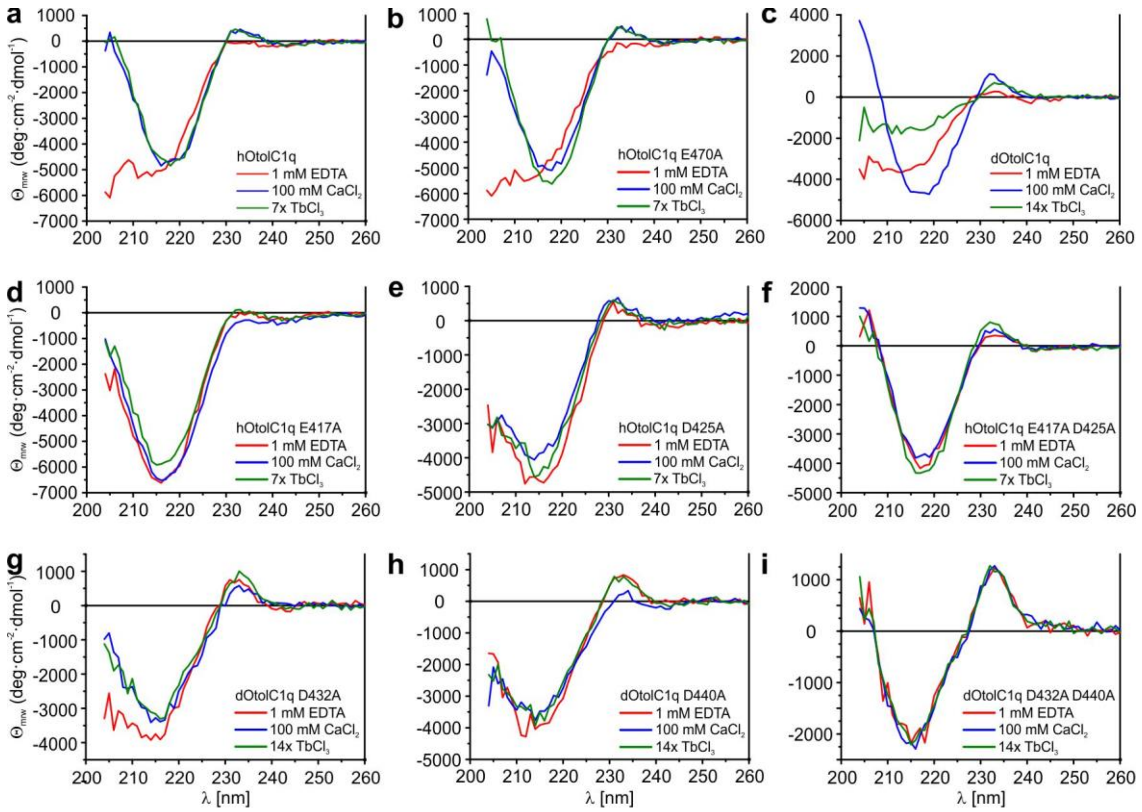

Suppl. Fig. 1 Changes in secondary structure of hOtolC1q, dOtolC1q and their mutants caused by Ca^{2+} and Tb^{3+}. Related to Fig. 3. Circular dichroism (CD) spectra were collected in the presence of 1 mM EDTA, 100 mM $CaCl_2$, 7-fold or 14-fold molar excess of $TbCl_3$. Protein concentration was 0.2 mg/mL

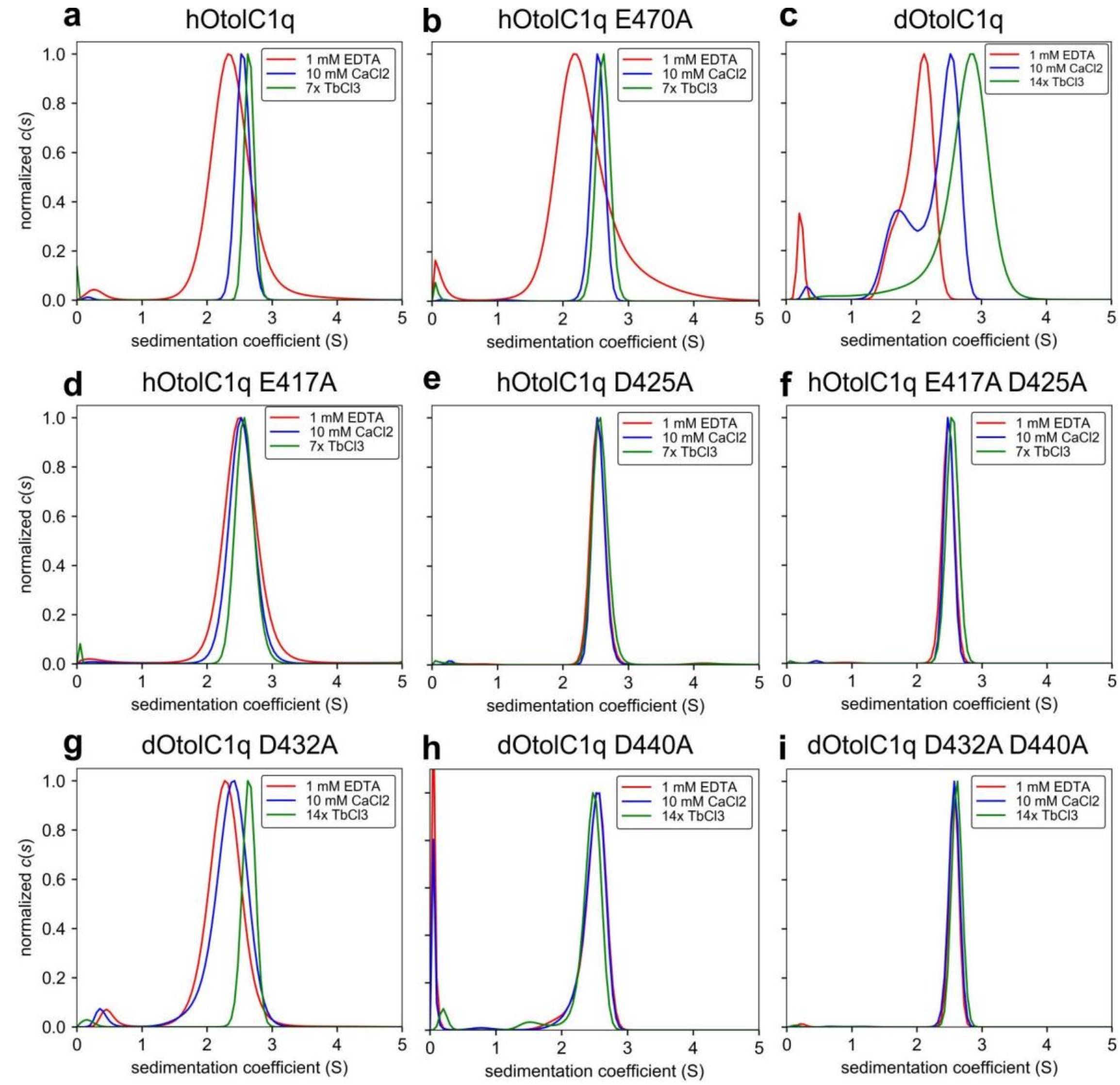

Suppl. Fig. 2 Normalized sedimentation coefficient distributions (*c*(*s*)) of hOtolC1q, dOtolC1q and their mutants in the presence of Ca^{2+} and Tb^{3+}. Related to Fig. 4. Sedimentation coefficient distributions were calculated for hOtolC1q, dOtolC1q and their mutants subjected to sedimentation velocity analytical ultracentrifugation in the presence of 1 mM EDTA, 10 mM $CaCl_2$, 7 or 14-fold molar excess of $TbCl_3$. The protein concentration was in the range 0.20 – 0.25 mg/mL

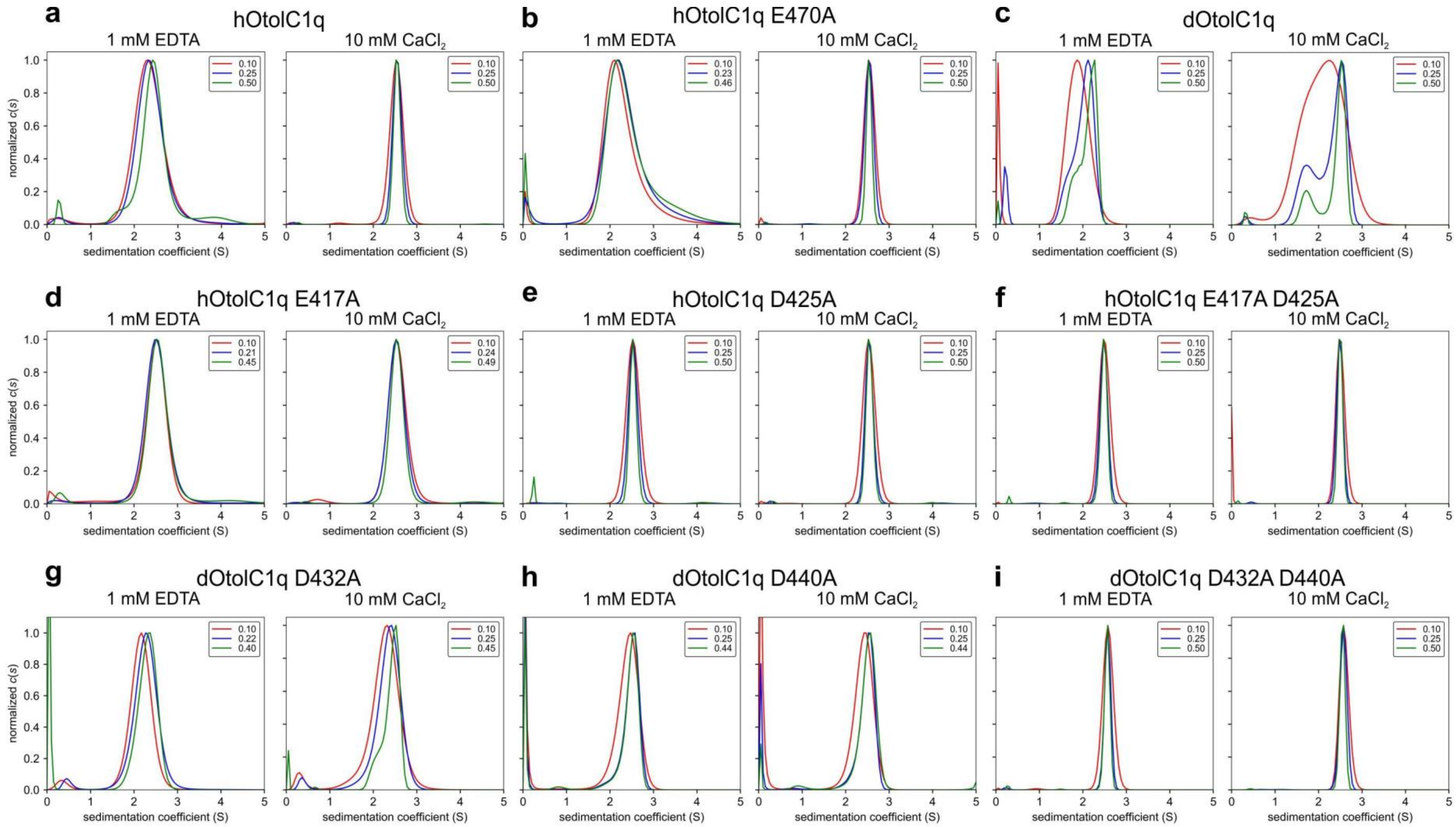

Suppl. Fig. 3 Concentration dependence of sedimentation coefficient distributions of hOtolC1q, dOtolC1q and their mutants. Related to Fig. 4. The *c*(*s*) distributions were calculated for various concentrations of hOtolC1q, dOtolC1q and their mutants subjected to sedimentation velocity analytical ultracentrifugation in the presence of 1 mM EDTA or 10 mM $CaCl_2$. Protein concentration was in the range of 0.1 – 0.5 mg/ml. The numbers in the figure panel legends indicate protein concentration in mg/mL

Suppl. Table 1 Summary of parameters derived from the sedimentation velocity analytical ultracentrifugation. Related to Fig. 4. Experiments were conducted in the presence of 1 mM EDTA, 10 mM $CaCl_2$ or appropriate molar excess of $TbCl_3$ (7x for hOtolC1q, 14x for dOtolC1q) at different protein concentrations. $\overline{s_{20,w}}$– weight-averaged sedimentation coefficient; MW_{app} – apparent molecular weight; f/f_0 – frictional ratio

Sample	*c* [mg/mL]	$\overline{s_{20,w}}$ [S]			MW_{app} [kDa]			f/f_0			RMSD		
		1 mM EDTA	10 mM $CaCl_2$	$TbCl_3$	1 mM EDTA	10 mM $CaCl_2$	$TbCl_3$	1 mM EDTA	10 mM $CaCl_2$	$TbCl_3$	1 mM EDTA	10 mM $CaCl_2$	$TbCl_3$
hOtolC1q	0.10	3.18	3.41	---	37.8	46.1	---	1.26	1.33	---	0.006018	0.005843	---
	0.25	3.22	3.45	3.58	39.8	45.6	50.3	1.29	1.31	1.35	0.006174	0.005934	0.007804
	0.50	3.36	3.45	---	41.9; 86.3	45.6	---	1.32	1.32	---	0.007856	0.006825	---
E417A	0.10	3.34	3.48	---	43.7	46.5	---	1.30	1.32	---	0.005520	0.005553	---
	0.24	3.40	3.43	3.50	43.1	44.3	46.1	1.28	1.30	1.31	0.006113	0.006040	0.007687
	0.49	3.54	3.49	---	44.2	44.2	---	1.28	1.29	---	0.006938	0.006956	---
D425A	0.10	3.41	3.42	---	45.6	47.0	---	1.32	1.34	---	0.005272	0.005607	---
	0.25	3.44	3.46	3.49	46.9	46.7	48.1	1.35	1.33	1.35	0.005790	0.005790	0.007117
	0.50	3.44	3.46	---	46.3	46.7	---	1.34	1.32	---	0.007282	0.006985	---
E417A D425A	0.10	3.37	3.39	---	47.4	51.0	---	1.37	1.43	---	0.006344	0.007504	---
	0.25	3.33	3.36	3.44	47.6	47.0	48.5	1.39	1.37	1.37	0.005976	0.005937	0.007389
	0.50	3.34	3.38	---	49.0	48.0	---	1.41	1.38	---	0.007780	0.007300	---
E470A	0.10	3.11	3.44	---	37.0	47.5	---	1.28	1.35	---	0.006334	0.006296	---
	0.23	3.21	3.41	3.53	40.2	47.7	47.6	1.31	1.36	1.32	0.006516	0.006498	0.007192
	0.46	3.35	3.43	---	42.1	47.3	---	1.29	1.35	---	0.007745	0.007942	---
dOtolC1q	0.10	2.54	2.81	---	25.8	31.7	---	1.27	1.32	---	0.006887	0.006101	---
	0.25	2.68	3.01	3.65	29.9	25.4; 42.5	46.2	1.33	1.37	1.31	0.006628	0.006551	0.007238
	0.50	2.82	3.13	---	32.6	26.4; 43.5	---	1.34	1.37	---	0.009029	0.008388	---
D432A	0.10	2.90	3.06	---	31.7	40.3	---	1.28	1.42	---	0.005332	0.005940	---
	0.25	3.05	3.16	3.55	37.8	41.1	48.0	1.37	1.39	1.38	0.006617	0.007261	0.007285
	0.45	3.09	3.23	---	35.8	39.1	---	1.30	1.32	---	0.006203	0.007543	---
D440A	0.10	3.21	3.23	---	40.0	39.7	---	1.34	1.33	---	0.005405	0.005163	---
	0.25	3.32	3.31	3.24	41.9	42.1	22.7; 44.0	1.34	1.34	1.40	0.005888	0.005863	0.007289
	0.44	3.27	3.35	---	41.9	44.1	---	1.35	1.37	---	0.006676	0.006600	---
D432A D440A	0.10	3.44	3.49	---	46.3	48.8	---	1.37	1.41	---	0.005691	0.005961	---
	0.25	3.44	3.44	3.51	46.4	46.8	47.6	1.38	1.38	1.38	0.005868	0.006315	0.007635
	0.50	3.42	3.41	---	46.6	46.6	---	1.39	1.39	---	0.007929	0.007789	---
